# Impacts of heifer postweaning residual feed intake classification on reproductive and performance measurements of first-, second-, and third-parity Angus beef females

**DOI:** 10.1093/tas/txab061

**Published:** 2021-04-05

**Authors:** Cory T Parsons, Julia M Dafoe, Samuel A Wyffels, Megan Van Emon, Timothy DelCurto, Darrin L Boss

**Affiliations:** 1 Northern Agricultural Research Center, Montana State University, Havre, MT 59501, USA; 2 Department of Animal and Range Sciences, Montana State University, Bozeman, MT 59717, USA

**Keywords:** beef cattle, heifer, parity, production, reproduction, residual feed intake (RFI)

## Abstract

This study evaluated heifer postweaning residual feed intake (**RFI**) classification on reproductive and performance measurements of first-, second-, and third-parity Angus beef females. We analyzed the annual, as well as, cumulative production of 347 Angus females from birth through weaning of their third calf. Heifer postweaning RFI was calculated as the actual dry matter intake minus the predicted dry matter intake based on the average daily gain of the contemporary group on an annual basis. Heifers were categorized based on RFI as either low (< −0.50 SD from mean), average (± 0.50 SD from mean), or high (> +0.50 SD from the mean) within year. There was no RFI × Parity interaction (*P* ≥ 0.14) observed for all production/reproduction traits except for conception rates (*P* = 0.02). Julian birth date of cows was influenced by RFI classification (*P* < 0.01) and displayed a quadratic (*P* = 0.02) effect with high RFI cows being born earlier in the calving season than average RFI cows (71.2 vs. 75.3 d), but did not differ from low RFI cows (74.0 vs. 75.3 d). Cow birth weight, weaning weight, as well as all other cow weight and body condition measurements were not influenced by RFI classification (*P* ≥ 0.14). As expected, there was a linear increase in cow body weight at weaning with increasing parity (*P* < 0.01). Cow RFI classification had no influence on progeny weaning weight, birth date, calving interval, or postpartum interval (*P* ≥ 0.15). Calf birth weights displayed a quadratic parity effect (*P* < 0.01) with first calf heifers having calves with lower birth weights than second- and third-parity calves. Calf 205-d adjusted weaning weights displayed a quadratic effect (*P* = 0.01) with first calf heifers weaning lighter calves than second- and third-parity cows. Weaning weight ratio displayed a linear decrease with increasing parity (*P* < 0.01). Cow conception probability displayed a linear tendency for pregnancy 2 (*P* = 0.09), and a quadratic tendency for pregnancy 4 (*P* = 0.07) as a function of RFI classification, but no effects were observed for pregnancy 1 and 3. Cow artificial insemination conception rates differed by year of pregnancy (*P* < 0.01), but not RFI classification (*P* = 0.81). In summary, heifer postweaning RFI classification had minimal effects on beef cattle production and reproductive efficiency.

## INTRODUCTION

Traditionally, feed efficiency of beef cattle has been expressed as the ratio of feed intake to body weight gained (feed to gain or gain to feed); however, selection for high growth rates inevitably increases the maintenance requirements, feed requirements, and intake of cattle, with subsequent higher environmental and feed costs (Okine et al. 2004). As cattle are selected for increased feed efficiency, managers often inadvertently select for larger mature sized cattle, thus, increasing the need for feed inputs in the future. In contrast, net feed efficiency (Byerly, 1941) or residual feed intake (**RFI**; Koch et al., 1963) is defined as the difference between an animal’s actual feed intake and its expected feed requirements for maintenance and growth. RFI is calculated as the difference between actual feed intake and predicted feed intake with negative or smaller values being more desirable than positive or larger values (Crews, 2005). Cattle that are more efficient than their contemporary group have a lower RFI value with feed intake being lower than predicted and cattle that are less efficient having a higher RFI value with feed intake being greater than predicted. RFI is an alternative measure of feed efficiency and is phenotypically independent of growth rate and body size (Crews, 2005; Arthur et al., 2008).

Multiple studies have reported that feed intake is a heritable trait in beef cattle (Koch et al., 1963; Fan et al., 1995; Nkrumah et al., 2007; Elzo et al., 2009; Mao et al., 2013; Retallick et al., 2017), with all studies estimating the heritability of feed intake in growing cattle between 0.28 and 0.44. Crowley et al. (2010) reported that RFI is moderately heritable at 0.45; therefore, selecting replacements with low RFI should produce energy-efficient cows and progeny. Estimates of RFI, as well as, feed efficiency are often used with bull test results and sire summaries and, in turn, recommended as a tool to select sires for beef production systems under the expectation of improved cow efficiency with low RFI selection pressure. However, information regarding the repeatability of RFI at different ages, at different stages of production, in different environments, and on different diets is limited (Black et al., 2013; Manafiazar et al. 2015; Freetly et al., 2020).

Biological mechanisms involved in beef cattle performance, as well as response to RFI classification, may interact with cow age, stage of production, and availability of feed resources (Sprinkle et al., 2020). Numerous factors may influence regulation of satiety in growing cattle, including composition of ration or diet. Freetly et al. (2020) stated that the signals for satiety in high concentrate rations may be associated with chemical signals, while those in forage-based diets are probably associated with gut fill. Previous research that has evaluated the impact of selection pressure for low RFI progeny is often inconsistent in respect to body condition, heifer maturity, and reproductive response. Randel and Welsh (2013) found that low RFI females were leaner, reached puberty at an older age, and calved later as heifers and subsequent calf crops. Similarly, Sprinkle et al. (2020) observed lower initial body condition score in 2-yr-old low RFI cows, but over the winter grazing period low RFI cows had less weight loss and concluded the study at a similar body condition as the high RFI cows. For their study, no differences were observed in body weight, yet weight and body condition losses were lower in the low RFI cattle. In contrast, Basarab and coworkers (2007) summarized 10 yr of performance records of dams that produced low, average, and high RFI progeny and concluded that dams who produced low RFI progeny had greater backfat (millimeter) and were in better condition prior to breeding than dams that produced high RFI progeny. No differences were observed in cow weights or calf 205-d adjusted weaning weights relative to RFI classification of the progeny (Basarab et al., 2007).

Most RFI studies are based on energy-dense diets focusing on feedlot performance (Lawrence et al., 2014), with limited information pertaining to RFI of cattle offered forage-based diets (Arthur et al., 2005; Black et al., 2013; Freetly et al., 2020) and even less information related to beef cows (Basarab et al., 2007; Meyer et al., 2008; Sprinkle et al., 2020). More research is needed related to the lifetime performance of low and high RFI cattle in forage-based rangeland environments aimed at increasing feed efficiency of western beef cattle operations (Manafiazar et al., 2015; Sprinkle et al., 2020). Black et al. (2013) stated the relationship between postweaning RFI and subsequent reproductive performance and longevity needs to be fully characterized to understand the impact RFI may have in a breeding scenario.

Therefore, objectives of the following research were to evaluate the relationship of heifer postweaning RFI classification to heifer performance data as calves and subsequent cow performance that includes reproductive efficiency, as well as, production traits, such as cow body weight, body condition, and calf weaning weights, in the absence of selection pressure based on RFI. Our premise was that this age group of cows (yearlings to 4.5 yr) would most likely to elicit production differences due to RFI classification of postweaning replacement heifers. We hypothesized that there is no difference between heifer postweaning RFI classification and reproductive performance or production parameters in beef cattle through their third weaned calf.

## MATERIALS AND METHODS

All procedures of this study were approved by the Montana State University Agricultural Animal Care and Use Committee (AACUC #2018-AA12).

### Heifer RFI Trials and Baseline Performance

Starting in 2008 to present, all Northern Agricultural Research Center (**NARC**) cattle were utilized in a heifer RFI trial for a minimum of 77 d on a forage-based ration provided in a GrowSafe system (GrowSafe DAQ 4000E; GrowSafe System Ltd., Airdrie, AB, Canada). Calves were weaned on pasture mid-September to early October each year and entered an RFI trial 60 to 75 d postweaning. On average, NARC retained 45–95 artificial insemination (**AI**) sired replacement heifers during the study period. Upon trial initiation, and completion, all heifers were weighed post feeding, on two consecutive days to record beginning and ending body weights, then again, every 28 d to record BW gain. A 7-d acclimation period preceded a 70-d feeding trial, while the GrowSafe system recorded individual daily feed intakes. All heifers had free access to 18 GrowSafe feed bunks and ad libitum access to water and forage-based diets, consisting of 30.4% corn silage, 41.1% grass hay, and 28.5% alfalfa on a dry mater basis, formulated to meet maintenance requirement for growing moderate frame beef heifers, 10.5% CP and 66.0% TDN (NASEM, 2016). Individual heifer postweaning RFI was calculated following previous parameters set forth by Archer et al. (1997), and Arthur et al. (2001). Heifers were categorized as either low (> −0.50 SD from mean; $\bar{x}$ = −0.05), average (± 0.50 SD from mean; $\bar{x}$ = 0.01), or high (< +0.50 SD from the mean; $\bar{x}$ = 0.48) within year (Table 1).

**Table 1.** Number of cows per RFI classification, production year, and 3 yr of calf production (*n* = 347)

	Yearling^1^			Parity 1			Parity 2			Parity 3		
Year Born	Low	Ave	High	Low	Ave	High	Low	Ave	High	Low	Ave	High
2010	26	36	33	22	31	33	19	26	29	17	21	21
2011	26	28	27	19	21	19	14	21	16	12	19	14
2012	22	18	23	10	10	7	9	8	17	8	6	15
2013	14	20	11	12	20	7	10	18	7	10	15	6
2014	20	26	17	14	21	12	13	21	11	13	18	7

Five years of heifer replacements (2010 to 2014) with 5 yr of performance data.

^1^Heifers were categorized as either low (> −0.50 SD from mean), or average (± 0.50 SD from mean) or high (< +0.50 SD from the mean) RFI classes.

### Subsequent Cow Performance

Mature Black Angus females (*n* = 347, Table 1) from the Montana State University NARC located in Havre Montana were utilized for this study. All mature cows were managed as one contemporary group, wintered at the NARC and summered at the Thackeray Ranch located south of Havre in the Bear Paw Mountains. All females were synchronized, and time AI in early June with exposure to cleanup bulls for an additional 45 d of natural service to calve during March and April the following year. Pregnancy diagnosis of all cows was performed via ultrasonography by a qualified Veterinarian at weaning each year with all calves being weaned early fall, mid-September to early October (150 to 10 d of age) and returned to the NARC Experiment Station. Calf 205-d weaning weights were adjusted for sex of calf and age of cow following BIF (2020) standards. Postpartum interval was determined by AI date for AI sired calves and back calculated based on calving date for natural service sired calves. Postpartum interval estimates assume that all RFI classifications and cow age have similar gestation length. All pregnant cows remained at the Thackeray Ranch grazing dormant late season forages through early January. Females that were culled from the herd were categorized as either being culled for reproductive reasons (open) or other (structure, disposition, calf died, cow died). For this study, individual animal was considered the experimental unit with RFI classification being the treatment.

### Statistical Analysis

Beef cattle production and reproduction parameters including: cow birth weight (**BW**), cow birth date, cow 205 day adjusted weaning weight, cow yearling weight, cow yearling body condition score (**BCS**), cow yearling pregnancy weight, cow yearling pregnancy BCS, cow weight at weaning, cow BCS at weaning, cow years in herd, cow calving interval, cow postpartum interval, calf BW, calf 205 day adjusted weaning weight, calf weaning weight ratio, and calf birth date were analyzed using ANOVA with a mixed model including RFI classification, calf number (parity), and the interaction of parity with RFI classification as fixed effects, and individual cow as the random effect. Data were plotted and natural log-transformed if needed to satisfy assumptions of normality and homogeneity of variance (cow BW). Log-transformed data were back transformed using the emmeans package in R (Lenth, 2019). Least-square means were separated using pairwise comparison when *P* < 0.05. For RFI and parity main effects, preplanned orthogonal polynomial contrasts were used to determine linear and quadratic effects. Beef cattle reproductive parameters eliciting a binomial response (conception rate, AI conception rate, present at 5 yr, culled due to pregnancy status) were analyzed using generalized linear models following a binomial distribution in an ANOVA framework. An α ≤ 0.05 was considered significant. All statistical analyses were performed in R (R Core Team, 2017).

## Results

### Heifer Baseline Performance

Julian birth date of retained heifers with differing RFI classification displayed a quadratic (*P* = 0.02) response with high RFI heifers being born earlier in the calving season (71.2 vs. 75.3 d) than average RFI heifers but not different than low RFI heifers (Table 2). In contrasts, heifer BW, heifer weaning weights, as well as body weight, and condition as yearlings and at pregnancy diagnosis did not differ across RFI classifications (*P* ≥ 0.14). Heifer weight and condition averaged 586 kg and 5.25, respectively, at pregnancy diagnosis for the first calf.

**Table 2.** The influence of RFI classification on birth weight, cow weight, body condition, and subsequent cow performance for 5 yr of retained heifers with 5 yr of performance data including three weaned calf crops

					*P*-value		
	RFI^1^				Preplanned contrast		
Category	Low	Ave	High	SE^2^	RFI	Linear	Quadratic
Heifer baseline data							
Birth wt. kg	37.4	36.0	35.5	2.55	0.27	0.27	0.76
Julian birth date^3^	74.0^ab^	75.3^b^	71.2^a^	0.99	< 0.01	0.05	0.02
Weaning wt., kg	239.3	237.4	240.6	5.39	0.14	0.77	0.43
Yearling wt., kg	488.1	501.2	504.4	13.70	0.17	0.08	0.49
Yearling BCS	5.8	5.8	5.9	0.06	0.55	0.59	0.35
Pregnancy wt., kg	586.1	585.7	586.6	4.22	0.83	0.93	0.89
Pregnancy BCS	5.2	5.3	5.3	0.05	0.91	0.20	0.57
Subsequent cow performance							
Calf birth wt., kg^4^	38.9	39.2	39.2	0.70	0.03	0.78	0.88
Calf weaning wt., kg	276.9	281.0	281.0	2.93	0.23	0.36	0.55
Calf weaning wt., ratio^5^	47.8	48.4	48.1	0.68	0.15	0.81	0.57
Calf Julian birth date	85.0	84.1	84.7	1.05	0.86	0.57	0.85
Calving interval, d	366.0	367.0	365.0	1.96	0.74	0.71	0.79
Postpartum interval, d	85.7	84.6	84.3	1.58	0.92	0.57	0.85

^1^Heifers were categorized as either low (> −0.50 SD from mean), average (± 0.50 SD from mean), or high (< +0.50 SD from the mean) RFI classes.

^2^Pooled standard error of the means.

^3^Means within rows lacking common superscript differ (*P* < 0.05).

^4^A post hoc means separation analysis showed no differences.

^5^Ratio of calf weaning weight to cow body weight.

### Subsequent Cow Performance

For most subsequent cow performance traits, parity (first, second, or third calf) did not interact with RFI classification (*P* > 0.10). Therefore, data are presented as main effects for RFI classification of dams (Table 2) or parity influences on beef cattle performance (Table 3). Calf BW differed relative to cow RFI classification (*P* = 0.03; Table 2), but a post hoc means separation analysis showed no differences with a range of only 38.9 to 39.2 kg. Calf weights at weaning and weaning rate ratio across three calf crops did not differ related to RFI classification of the dams (*P* ≥ 0.55) averaging 280 kg and 48%, respectively. In addition, calf Julian birth date, calving interval, and postpartum interval were not influenced by RFI classification of the dam (*P ≥* 0.79) averaging 83.7, 366, and 84.8 d, respectively.

**Table 3.** The influence of parity on beef cow performance, and reproductive parameters for 5 yr of retained heifers with 5 yr of performance data including three weaned calf crops

					*P*-value		
	Parity					Preplanned contrast	
Category	1	2	3	SE^2^	Parity	Linear	Quadratic
Cow wt. at weaning, kg^3^	537.5^a^	591.5^b^	627.3^c^	4.34	< 0.01	< 0.01	0.11
Cow BCS at weaning	5.2	5.2	5.4	0.05	0.28	< 0.01	0.04
Calf birth wt., kg	34.1^a^	40.9^b^	42.4^b^	0.66	< 0.01	< 0.01	< 0.01
Calf weaning wt., kg	271.7^a^	285.8^b^	281.2^b^	2.79	0.03	0.02	0.01
Calf wean wt. ratio	50.8^a^	48.2^b^	45.3^c^	0.69	< 0.01	< 0.01	0.88
Calf Julian birth date	84.5	84.4	84.9	1.07	0.27	0.82	0.80
Calving interval, days	NA	366.0	366.0	1.60	0.22	NA	NA
Postpartum interval, days	87.4^a^	86.6^a^	80.6^b^	1.57	0.02	< 0.01	0.16

^1^Heifers were categorized as either low (> −0.50 SD from mean), average (± 0.50 SD from mean), or high (< +0.50 SD from the mean) RFI classes.

^2^Pooled standard error of the means.

^3^Means within rows lacking common superscript differ (*P* < 0.05).

As expected, parity number did influence cow weight at pregnancy (*P* < 0.01; Table 3), with third-parity pregnancy determination weights being heavier than the second and first, and second-parity pregnancy determination weights being heavier than first parity. Calf BW differed by parity (*P* < 0.01) with first parity calves being lighter than second- and third-parity calves. Calf 205-d adjusted weaning weights displayed a quadratic effect (*P* = 0.01) with first calf heifers weaning lighter calves than second- and third-parity cows. In contrast, weaning weight ratio displayed a linear decrease with increasing parity (*P* < 0.01) ranging from 50.8% for first-parity heifers to 45.3% for third-parity cows. Cow postpartum interval displayed a linear effect (*P* < 0.01) with third-parity females having fewer days between calving and conception than first- and second-parity females. Calf Julian birth dates were not influenced by parity (*P* = 0.27) and averaged 84.6 d.

Cow conception rates following a 45-day breeding season (Table 4) exhibited an RFI × parity interaction (*P* = 0.02). Within breeding season, second calf cows tended (*P* = 0.09) to display a linear increase in conception with increasing RFI classification. In contrast, cow conception probability for the fourth calf tended (*P* < 0.07) to display a quadratic response with average RFI cows having lower conception than low or high RFI cows. Pregnancy rates of yearling heifers and second calf cows did not differ among RFI classifications (*P* > 0.10) averaging 87.8 and 91.7 %, respectively.

**Table 4.** The influence of RFI classification of heifers on subsequent beef cow conception probability and AI probability and cow longevity spanning four-breeding seasons and three weaned calf crops

	RFI^1^				*P*-value			Preplanned contrast	
Category	Low	Ave	High	SE^2^	RFI	Pregnancy	RFI × Pregnancy	Linear	Quadratic
Cow conception probability, %	94.0	93.7	93.3	1.8	0.75	0.05	0.02		
Pregnancy 1	83.6	91.3	88.6	3.4				0.11	0.14
Pregnancy 2	91.0	98.2	96.5	2.3				0.09	0.14
Pregnancy 3	95.9	91.2	87.9	3.2				0.15	0.68
Pregnancy 4	98.0	89.3	96.0	3.1				0.70	0.07
Cow AI conception probability, %	55.0	59.5	59.5	3.3	0.81	<0.01	0.32	0.36	0.55
Years in herd^3^	4.5	4.3	4.7	0.2	0.07	NA	NA	0.23	0.08
Present at 5 yr, %	50.9	53.1	51.4	4.6	0.94	NA	NA	0.95	0.72
Culled due to pregnancy status, %	37.0	29.7	37.8	4.4	0.34	NA	NA	0.90	0.14

^1^Heifers were categorized as either low (> −0.50 SD from mean), average (± 0.50 SD from mean), or high (< +0.50 SD from the mean) RFI classes.

^2^Pooled standard error of the means.

^3^Length of time cows remained in herd over the 5-yr study period.

Cows conceiving to AI did not differ by RFI classification (*P* = 0.81), with AI conception averaging 58% across all RFI classifications of the dams. However, AI conception rates were influenced by parity (*P* < 0.01) with first and third calf cows having lower conception rates (48.3 and 54.3, respectively) than second- and fourth-parity calf cows (62.4 and 66.5, respectively). RFI classification displayed a quadratic tendency (*P* = 0.08) with cow longevity (years in herd), with high RFI cows having greater longevity than average RFI cows, with low RFI cows being intermediate. Heifer RFI classification did not affect (*P* = 0.94) probability of remaining in the herd at the age of 5 averaging 4.5 yr, and 51.2%, respectively. In addition, the percentage of females culled because of pregnancy status did not differ by RFI classification (*P* = 0.14) averaging 34.8% over the first 5 yr of production.

## DISCUSSION

Our study evaluated the production records of 347 replacement heifers for their first five production years based on a heifer postweaning RFI classification. We did see differences in Julian date at calving with low RFI heifers being born later in the calving period than high RFI heifers. However, we did not see any impact of RFI on birth date of progeny. In contrast, Randel and Welsh (2013) observed later calving dates for low RFI cattle as heifers and subsequent calving dates as cows. In addition, no differences were detected for cow body condition at breeding or weaning for three consecutive calf crops.

Based on performance traits, such as body weight, body condition score, calf BWs, and longevity in the herd, our results suggest that RFI rankings did not influence cow calf production parameters in our foraged-based environment. This is in general agreement with Manafiazar et al. (2016), which states that RFI has zero phenotypic correlation with production traits and near-zero genetic correlation to production traits in Dairy cows. However, it is important to note that no selection pressure was placed on RFI while selecting replacement heifers, AI sires, and cleanup bulls during this study period.

Previous research has reported cow body weight and calf weaning weights over multiple production cycles were similar for cows classified as Low, Medium, and High RFI (Basarab et al. 2007). Our results agree with this as we observed no difference due to RFI classification of heifers on subsequent body weight or weaning weights of young cows over three calf crops weaned. This would be expected as RFI has been demonstrated to be independent of body weight and growth (Arthur et al. 2005; Crews, 2005, Manafiazar et al., 2016), which concluded that phenotypic and genetic relationship of RFI on body weight is near zero and is consistent from postweaning through maturity. Most of the differing performance characteristics observed in these studies were attributed to age (parity, or pregnancy number) and would be expected to increase as average annual body weight, BWs, adjusted 205-d weaning weights, increase with increasing cow age (Renquist et al. 2006).

While RFI may present an opportunity to reduce feed costs, mixed results exist regarding the effect of RFI classification on reproductive performance (Arthur et al., 2011; Basarab et al., 2011; Blair et al., 2013). Damiran et al. (2018) reported a tendency for low RFI heifers to exhibit lower pregnancy rates than high RFI heifers, with fewer low RFI heifers calving in the first cycle compared to high RFI heifers. Arthur et al. (2005) and Blair et al. (2013) reported no differences between high and low RFI lines for pregnancy rate. In contrast, Randel and Welsh (2013), in a review, stated selection for low RFI results in selection of leaner heifers that reach puberty later and concluded that selection for low RFI may impair reproductive efficiency. This is supported by Arthur et al. (2005), who reported that low RFI cows calved 8 d later than high RFI cows, with the progeny of low RFI cows calving 5 to 6 d later than high RFI cows.

In our study, AI conception rates did not differ by RFI classification averaging 58% conception across all RFI classifications. However, AI conception rates were influenced by parity with first and third calf cows having lower conception rates (48.3 and 54.3, respectively) than second and fourth calf cows (62.4 and 66.5, respectively). Similar to Arthur et al. (2005) we observed an RFI effect on Julian birth date with high RFI cows being born earlier in the calving season than average RFI cows. However, there was no RFI effect on subsequent calf Julian birth date.

While our study did not observe substantial difference in beef cattle performance through the weaning of the third calf, this does not preclude potential benefits of RFI selection for cattle that eat less forage and/or utilize rangeland areas more efficiently. In fact, the real benefit of selecting for low RFI cattle may relate to intake per unit of production rather than overall production traits per se. In one of the few studies that evaluated forage intake as a function of RFI classification, Lawrence et al. (2014) reported no difference in haylage or pasture forage intake as a result of RFI estimates. In contrast, Basarab et al. (2007) reported that cows that produce low RFI progeny consume less feed than cows that produce high RFI progeny based on a postweaning intake trial in confinement with a barley silage, barley straw-based ration. In addition, in a sagebrush steppe rangeland environment, Sprinkle et al. (2020) found the low RFI cows lost less weight and body condition than high RFI cows during a fall to early winter grazing period. The authors suggested that low RFI cattle had lower maintenance energy requirements or were more efficient grazers in the limited nutrition winter range environment as compared to high RFI cattle. However, no differences were observed in distance traveled or grazing behavior among low and high RFI cows (Sprinkle et al., 2020). Although difficult to assess, the actual benefit of using RFI classification to select cattle for environmental fitness may hinge on the effect of RFI on forage intake across diverse forage base systems that demonstrate a range of forage quantity, as well as, quality. Black et al. (2013) reported that while RFI for postweaning and lactation phases do not appear to be related, selection of the most feed-efficient heifers based on postweaning RFI may have economic implications by reducing feed costs and maintaining similar cow performance throughout lactation. Additionally, Damiran et al. (2018) indicated low RFI heifers had lower feed costs than high RFI heifers. Similarly, Manafiazar et al. (2015) reported that low RFI yearling Bos taurus heifers grazing irrigated improved pastures in Alberta, Canada consumed less forage than high RFI heifers. If post weaning heifer RFI classifications are a good predictor of subsequent cow intake and the conversion of forage to calf production, selection of low RFI heifers would be beneficial.

## IMPLICATIONS

Classification of RFI for replacement heifers had little to no effect on subsequent beef cattle production and reproductive efficiency through the weaning of the third calf. Subtle differences were denoted for cow Julian birth dates based on RFI classification and conception of first calf-heifers categorized as low RFI. However, all other production parameters were similar across RFI classifications of dams, with no difference in kg of calves weaned and longevity traits of the cows. Therefore, our study suggests that selection for low RFI females would not affect overall herd productivity of cattle on foraged-based production systems. However, further research is needed to investigate the relationship of heifer postweaning RFI classification on subsequent intake of forage base diets that would likely include pasture utilization, foraging behavior, supplement intake behavior, as well as, utilization and distribution in extensive rangeland environments. Research is limited in respect to the relationship of RFI classification and cow intake, as well as, cow age/intake interactions in forage-based production systems.
